# Effect of Fasting on Contrast Sensitivity in Healthy Males

**DOI:** 10.18502/jovr.v14i3.4789

**Published:** 2019-07-18

**Authors:** Zarife Ekici Gok, Abuzer Gunduz, Cem Cankaya

**Affiliations:** Inonu University School of Medicine, Department of Ophthalmology, Malatya, Turkey

**Keywords:** Contrast Sensitivity, Fasting, Ramadan Fasting

## Abstract

**Purpose:**

To evaluate the effect of fasting on contrast sensitivity (CS) in healthy male individuals during the month of Ramadan.

**Methods:**

This study included 45 healthy male individuals, aged between 20 and 40 years, working in the same environment. Functional acuity contrast testing (F.A.C.T) was performed using the Optec 6500 vision testing system. Measurements taken during a state of satiety one week before Ramadan were compared with those taken a minimum of 12 hours after the start of fasting in the first and fourth weeks of Ramadan.

**Results:**

Contrast sensitivity (CS) was increased at the spatial frequency of three cycles per degree (cpd) at the end of the first week of Ramadan in comparison to the CS measured before Ramadan (P = 0.03). The mean CS values were increased at the spatial frequencies of 3 and 12 cpd at the end of the last week of Ramadan in comparison to the mean values measured before Ramadan (P = 0.01 for both).

**Conclusion:**

Although we found statistically significant increases in CS at certain frequencies, we can conclude that Ramadan fasting has no negative effects on CS.

##  INTRODUCTION

Ramadan is a holy month in the Islamic calendar during which Muslims avoid certain behaviors, such as eating and drinking, from sunrise to sunset due to their religious beliefs. ^[1,2]^. 

The contrast sensitivity (CS) test measures a patient’s ability to perceive large, medium-sized, and small symbols under various contrast conditions. The test examines the finer details of vision that cannot be measured with a visual acuity test and can also detect the visual losses that cannot be detected with the Snellen test. Typically, the test is used to investigate visual function and early signs of eye disorders.^[3-5]^

The majority of the studies investigating the effects of religious fasting on ocular parameters are related to anterior segment parameters, glaucoma, and tear film stability. Theoretically, the restriction of fluid intake during the day may lead to the deterioration of tear film stability, and the shrinkage of the anterior chamber structures, including the lens and cornea. It is known that anterior chamber depth, refractive status of the eye, corneal curvature, as well as the tear film layer can be affected by dehydration.^[6,7,8,9,10]^

To the best of our knowledge, there are no studies fully investigating the effect of Ramadan fasting on CS in the literature, and therefore, in the current study, we aimed to address this gap in knowledge.

##  METHODS

This prospective study was performed at the Department of Ophthalmology in the Inonu University Medical Faculty on 45 healthy, male volunteers aged between 20 and 40 years who were working in the same environment and fasting for Ramadan between 27 June and 27 July in the summer of 2014. Females were excluded from the study as the menstrual cycle leads to disruption in Ramadan fasting. All participants gave informed and signed consent before the study, and the tenets of the Declaration of Helsinki were followed. Ethics approval was obtained from the Malatya Ethics Committee.

A full ophthalmologic examination was conducted by the same ophthalmologist in all cases before Ramadan. Corrected and uncorrected visual acuity was evaluated with the Snellen chart. All cases underwent biomicroscopic examinations, intraocular pressure (IOP) measurements using the Goldmann applanation tonometer, and fundus examinations. Cases that had best corrected visual acuity (BCVA) scores of 20/20 in both eyes and had no ocular or systemic pathology were included in the study. All cases were first informed about the CS test (grating test), which was then performed after a full ophthalmologic examination. This test, together with refraction and visual acuity tests, were performed right before Ramadan and at the end of the first and fourth weeks of Ramadan. Baseline measurements were obtained in a state of satiety (between 3 and 5 PM) a week before Ramadan. Measurements taken after the start of Ramadan fasting were obtained 12 to 14 hours after the cases began fasting. The mean duration of Ramadan fasting was 16.5 hours during the year in which we were performing the study.

###  Contrast sensitivity measurement

Spatial contrast was evaluated with the FACT (Functional Acuity Contrast Test, Stereo Optical Co., Chicago, IL, USA) panel. This panel includes five spatial frequencies (1.5, 3, 6, 12, and 18 cycles per degree [cpd]) of nine sinusoidal grating patches. The contrast of the grating patch decreases logarithmically from left to right. Test results were presented in log10 units of CS (logCS).

###  Statistical analyses

For statistical analyses of the data, the SPSS software for Windows, version 17.0 (Statistical Package for Social Sciences) was used. Mean, standard deviation, minimum, and maximum values were calculated for each parameter and as the variables were not normally distributed, the non-parametric Wilcoxon signed-rank test was used for comparison purposes. *P*-value < 0.05 was considered as statistically significant.

##  RESULTS

The mean age was 28.75 ± 8.42 years (range, 20 - 39 years) and the BCVA was 20/20 in both eyes of all cases. Biomicroscopic and fundus examinations were bilaterally normal and IOP measurements were within normal limits in all cases.

Of the total 45 cases, 4.4% had emmetropia, 20% had hyperopia (range of +0.25 to +2.00 D), 68.8% had myopia (range of –3.00 to –0.25 D), and 6.6% had astigmatism (range of –2.00 to –0.25 D). The mean spherical equivalent was –0.22 ± 0.58 D.

The BCVA was 20/20 bilaterally when measured before Ramadan and in the first and fourth weeks of Ramadan. No statistically significant changes were observed in terms of refractive errors and visual acuity (*P* > 0.05).

After measurements were taken at five spatial frequencies, the mean values equivalent to each grating, based on spatial frequencies, were recorded [Table 1].

**Table 1:** The contrast values equivalent to each grating according to spatial frequency


**Spatial frequency (cpd)**	**1st level**	**2nd level**	**3rd level**	**4th level**	**5th level**	**6th level**	**7th level**	**8th level**	**9th level**
A (1.5)	7	9	13	18	25	36	50	71	100
B (3)	10	15	20	29	40	57	80	114	160
C (6)	12	16	23	33	45	64	90	128	180
D (12)	8	11	15	22	30	43	60	85	120
E (18)	4	6	8	12	17	23	33	46	65
	
	
cpd, cycles per degree									

**Figure 1:**
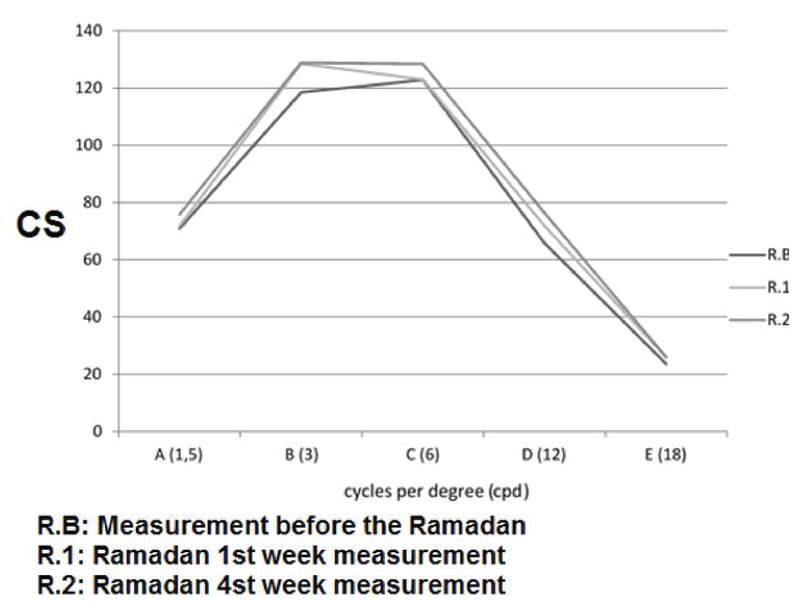
Comparison of the mean contrast sensitivity (CS) measurements.

The CS was increased at the spatial frequency of three cpd at the end of the first week of Ramadan in comparison to the value recorded before Ramadan (*P* = 0.03). The mean CS values were increased at spatial frequencies of 3 and 12 cpd at the end of the last week of Ramadan in comparison to before Ramadan (*P* = 0.01 for both). No statistically significant differences were observed in CS values at other spatial frequencies [Figure 1, Tables 2 and 3].

**Table 2:** The comparison of contrast sensitivity measurements conducted before the Ramadan and in the first week


**Spatial frequency (cpd)**	**Measurements before the Ramadan (mean ± SD)**	**Ramadan first week measurements (mean ± SD)**	**** ***P*** **-value**
A (1.5)	70.87 ± 22.52	71.78 ± 21.58	0.95
B (3)	118.45 ± 22.22	128.52 ± 24.25	0.03*
C (6)	122.82±30.38	123.02 ± 31.40	0.75
D (12)	65.85 ± 25.55	71.89 ± 26.22	0.29
E (18)	23.57 ± 1.42	25.91 ± 14.43	0.28
	
	
cpd, cycles per degree; SD, standard deviation			

**Table:** The comparison of contrast sensitivity measurements conducted before the Ramadan and at the fourth week of Ramadan

**Spatial frequency (cpd)**	**Measurement before Ramadan (mean ± SD)**	**Ramadan fourth week measurement (mean ± SD)**	**** ***P*** **-value**
A (1.5)	70.87 ± 22.52	75.75 ± 21.88	0.17
B (3)	118.45 ± 22.22	128.84 ± 24.42	0.01*
C (6)	122.82 ± 30.38	128.40 ± 26.18	0.49
D (12)	65.85 ± 25.55	76.35 ± 27.61	0.01*
E (18)	23.57 ± 1.42	25.95 ± 13.20	0.13
	
	
cpd, cycles per degree; SD, standard deviation			

**Spatial frequency (cpd)**	**Ramadan first week measurement (mean ± SD)**	**Ramadan fourth week measurement (mean ± SD)**	**** ***P*** **-value**
A (1.5)	71.78 ± 21.58	75.75 ± 21.88	0.10
B (3)	128.52 ± 24.25	128.84 ± 24.42	0.91
C (6)	123.02 ± 31.40	128.40 ± 26.18	0.47
D (12)	71.89 ± 26.22	76.35 ± 27.61	0.83
E (18)	25.91 ± 14.43	25.95 ± 13.20	0.76
cpd, cycles per degree; SD, standard deviation			

Although there was an increase in CS measurement values conducted at all spatial frequencies during the first and fourth weeks of Ramadan, none of these were statistically significant (*P* > 0.05); [Table 4].

##  DISCUSSION

Our findings show that CS was increased at the spatial frequency of three cpd at the end of the first week of Ramadan in comparison to CS measured before Ramadan. In addition, the mean CS values were increased at the spatial frequencies of 3 and 12 cpd at the end of the last week of Ramadan compared to the values measured before Ramadan. We found statistically significant alterations at certain spatial frequencies in the CS test during Ramadan; however, in general, these alterations likely have no significant effect. The effect of fasting during Ramadan on ocular parameters has been evaluated in many studies;^[9,10]^ however, CS has not yet been studied.

When considering CS function in the human eye from very low spatial frequencies to very high spatial frequencies, in general, we observe a continuous decrease. CS in a normal eye increases from the lowest frequencies to approximately six cpd and then decreases with higher frequencies. This decrease in CS is due to the diffraction and aberration that makes visual details more difficult to perceive.^[3,4,5]^ This pattern in CS function is due to the programming of the retinal-brain visual processing system.^[11,12]^ We found statistically insignificant increases at all frequencies and a statistically significant increase at three cpd in CS measurements in the first week of Ramadan compared with the measurements made before Ramadan. We also found statistically significant increases at 3 and 12 cpd and statistically insignificant increases at other frequencies in the measurements in the last week of the Ramadan.

While the CS decreases gradually at frequencies higher than six cpd, our study showed a statistically significant increase at 12 cpd; however, statistically significant increases were not observed at all other frequencies.

Fasting leads to changes in physiological parameters and these changes affect the ocular system.^[7]^ An increase in free fatty acid, norepinephrine, and cortisol concentrations occurs as a result of a decrease in insulin secretion and an increase in both glucagon levels and sympathetic activity.^[8]^ Retinal hyperperfusion and increased IOP due to these hormones have been reported as the basic effects of Ramadan fasting on ocular parameters.^[9,10]^

In a study conducted by Koktekir et al, fasting was found to significantly decrease tear production and increase tear osmolarity but have no effect on corneal topographic parameters or produce ocular aberrations.^[13]^ In another study conducted by Selver et al, no significant differences were detected in any of the anterior segment parameters, visual acuity measurements, or IOP measurements during Ramadan fasting.^[14]^ Kerimoglu et al^[15]^ demonstrated that fasting might lead to a decrease in IOP; however, in the study conducted by Kayikcioglu et al,^[16]^ this correlation was not observed. Refractive status, lens thickness, or corneal curvature changes may occur as a result of dehydration during the Ramadan period. Changes may occur in the refractive index of the dehydrated vitreous and this may affect the axial length, resulting in either a very small or no refractive change. Although the anterior chamber depth is affected by fluctuations in the hydration status of the body, a long period of dehydration is required for changes in axial length to manifest.^[17]^ According to the results of these studies, alterations in the anterior chamber parameters could also impair CS functions. In our study, we also evaluated refractive status and visual acuity of the subjects; however, no statistically significant change was observed in any of the measurements (*P* > 0.05). Our cases demonstrated no visual acuity loss.

One of the main limitations of our study was the number of participants. A larger cohort of participants may provide more supporting information on the effect of Ramadan fasting on CS function. The other limitation of our study was the lack of a control group, as we compared CS measurements within the same patient population. Our results may have been more valuable upon inclusion of a control group.

In conclusion, we showed that Ramadan fasting has no negative effects on visual acuity and CS. In fact, we found a statistically significant increase in CS at some frequencies using the CS test.

We believe that additional studies need to be performed to obtain more supporting information on the effects of Ramadan fasting on the eye and ocular CS function. We think that physicians working in countries with sizeable Muslim populations should be aware of the physiological effects of Ramadan fasting, particularly pertaining to various ocular problems and treatments.

##  Financial Support and Sponsorship

Nil.

##  Conflicts of Interest

There are no conflicts of interest.
